# Genetic Diversity of Namibian* Pennisetum glaucum* (L.) R. BR. (Pearl Millet) Landraces Analyzed by SSR and Morphological Markers

**DOI:** 10.1155/2016/1439739

**Published:** 2016-06-28

**Authors:** Billy McBenedict, Percy Chimwamurombe, Ezekeil Kwembeya, Gillian Maggs-Kölling

**Affiliations:** ^1^Department of Biological Sciences, University of Namibia, 340 Mandume Ndemufayo Avenue, Windhoek, Namibia; ^2^Gobabeb Research and Training Centre, Namib Naukluft Park, Walvis Bay, Namibia

## Abstract

Current* Pennisetum glaucum* (L.) R. BR. cultivars in Namibia have overall poor performance posing a threat to the nation's food security because this crop is staple for over 70% of the Namibian population. The crop suffers from undesirable production traits such as susceptibility to diseases, low yield, and prolonged reproductive cycle. This study aimed to understand the genetic diversity of the crop in Namibia by simple sequence repeats (SSRs) and morphology analysis. A total of 1441 genotypes were collected from the National Gene Bank representing all the Namibian landraces. A sample of 96 genotypes was further analyzed by SSR using Shannon-Wiener diversity index and revealed a value of 0.45 indicating low genetic diversity. Ordination using Principal Coordinate Analysis (PCoA) on SSR data confirmed clusters generated by UPGMA for the 96* P. glaucum* accessions. UPGMA phenograms of 29 morphological characterized genotypes were generated for SSR and morphology data and the two trees revealed 78% resemblance. Lodging susceptibility, tillering attitude, spike density, fodder yield potential, early vigour, and spike shape were the phenotypic characters upon which some clusters were based in both datasets. It is recommended that efforts should be made to widen the current gene pool in Namibia.

## 1. Introduction

Genetic diversity is defined as a variety of genes in a given species which are important for adaptation and conservation of desired traits and drives survival during natural selection [1]. Variations can occur in many forms such as the number and structure of chromosomes, amount of DNA found in cells, and dissimilarities in the DNA sequence. Genetic diversity occurs as an outcome of mutations, recombination, genetic drift, migration, and selection. Genetic diversity is an important aspect in the exploration of plant genetic resources. These resources have proved to be beneficial in conservation genetics and have potential applications in crop improvement [2]. Crop improvement programs are successfully implemented with the knowledge of the gene pool of the crop under investigation. This information is important because both the environment and genes play an important role in the phenotype of an organism [3]. The gene pool can be assessed for genetic variation by scrutinizing the phenotype and genotype of an organism. The phenotype of an individual is a product of the collective interaction between the environment and the individual's genes while the genotype is the actual genetic makeup of an organism [4].

*Pennisetum glaucum* is a multipurpose cereal widely cultivated in different parts of the world for grain, stover, and green fodder on about 27 million hectares of land, primarily in Asia and Africa [5]. It is the sixth most important cereal crop annually produced in the world after wheat, rice, maize, barley, and sorghum [3]. Pearl millet is a highly nutritious staple food crop widely grown in different regions of Namibia, namely, Zambezi, Kavango East, Kavango West, Otjozondjupa, Oshikoto, Kunene, Omusati, Ohangwena, and Oshana. Among other uses, the crop is used to brew opaque beer and to cook traditional scones which are also fed to chickens [6].* P. glaucum* accessions have been collected, morphologically characterized, and stored in the National Plant Genetic Resource Centre of Namibia (the Gene Bank of Namibia) in the past years. However, there have been no studies on the molecular characterization of the Namibian* P. glaucum* genotypes. This study therefore aimed to determine the genetic diversity of* P. glaucum* landraces by means of microsatellites markers.

## 2. Materials and Methods

### 2.1. Germplasm Collection

All the accessions which amounted to 1441 landraces were obtained from the Namibian National Plant Genetic Resource Centre (NPGRC) and used in the present study. These accessions were collected in 1991 by the International Crops Research Institute of Semi-Arid Tropics (ICRISAT) and have been multiplied and characterized for gene banking purposes. From a total of 1441 accessions, the sample size of 96 landraces was calculated as described by literature (Table 2) [7]. Sampling was done in such a way that all the regions from which the* P. glaucum* accessions were collected were represented (Table 1) in the calculated sample size. This was achieved by random sampling from each region based on proportionate calculations.

### 2.2. Deoxyribonucleic Acid (DNA) Extraction

Genomic DNA was extracted using the cetyltrimethylammonium bromide (CTAB/chloroform-isoamyl alcohol) protocol as documented [8]. The DNA was run using 0.8% agarose gel and the DNA concentration was determined using a NanoDrop 2000c spectrophotometer (Thermo Scientific, Massachusetts, USA). Samples (96) of DNA were amplified in 25 *μ*L PCR reaction volumes using primers that revealed polymorphism based on agarose gel patterns.

### 2.3. Primer Screening

A total of 25 SSR primers described by Neto et al. [9] shown in Table 1 were obtained from Inqaba Biotechnical Industries and were screened for polymorphism. This was done by randomly selecting 2 DNA samples from each of the regions (Zambezi, Karas, Kunene, Ohangwena, Okavango [East and West], Omusati, Oshana, Oshikoto, and Otjozondjupa) involved in the origin of all the accessions. The primers were dissolved in nuclease-free water to make 100 *μ*M stock solutions. The primer stock solutions were then diluted to form 20 *μ*M aliquots of each primer pair. Extracted DNA samples with a concentration of 20–250 *μ*g/*μ*L were diluted to 10 ng of DNA per *μ*L for PCR amplification. A total of 18 DNA samples were then amplified in a 25 *μ*L PCR reaction volume comprising 12.5 *μ*L of 2x master mix, 1 *μ*L of template DNA, 1 *μ*L of a 1 *μ*M concentration forward SSR primer, 1 *μ*L of a 1 *μ*M concentration reverse SSR primer, and 9.5 *μ*L of nuclease-free water with thermocycling conditions of initial denaturation step at 95°C for 4 min, followed by 35 cycles of denaturation at 94°C for 1 min, annealing ranged between 57°C and 62°C which was primer dependent for 1 min, and elongation at 72°C for 1 min. Final elongation was performed at 72°C for 4 min and held at 4°C. 2% agarose gel electrophoresis with a 1 kb generule, DNA ladder to determine the pair of primers that displayed polymorphism or monomorphism based on the amplification product band patterns of separation on the gel from the different DNA templates. The outcome of the screening was described as monomorphic, polymorphic, or unable to amplify. The PCR was then conducted with the actual samples but using the primers that displayed a higher level of polymorphism.

### 2.4. Microsatellite Analysis

Of the 25 primer sets tested, six (6) primer sets (3005, 3016, 3020, 3022, 3035, and 3039) displayed polymorphism and were used to amplify microsatellites loci by combining 1 *μ*L of template DNA, 1 *μ*L of a 1 *μ*M concentration forward SSR primer, 1 *μ*L of a 1 *μ*M concentration reverse SSR primer, 9.5 *μ*L of nuclease-free water, and 12.5 *μ*L of 2x Dream* Taq* master mix which contained Dream* Taq* DNA Polymerase, 2x Dream* Taq* buffer, deoxyadenosine triphosphate (dATP), deoxycytidine triphosphate (dCTP), deoxyguanosine triphosphate (dGTP), and deoxythymidine triphosphate (dTTP) of 0.4 mM each and 0.4 mM MgCl_2_. The polymerase chain reaction (PCR) conditions were 35 cycles of initial denaturation temperature of 94°C for 3 min, denaturation temperature at 94°C for 1 min, primer annealing for 1 min at respective primer annealing temperatures, and 72°C extension temperature for 1 min. The final extension was then performed at 72°C for 4 min and lastly held at 4°C.

### 2.5. Data Analysis

The Polymorphism Information Content (PIC) values for each SSR primer were estimated by determining the frequency of alleles per locus as described by Sharma et al. [10] using the formula, PIC = 1 − ∑*x*_*i*_^2^, where *x*_*i*_ is the relative frequency of the *i*th allele of the SSR loci (Table 3). Markers were designated as being informative when PIC was ≥ 0.5. The most informative primers (3005, 3035, and 3039) were then used for subsequent genetic diversity calculations since they gave a PIC value greater than 0.5. Primers 3016 and 3020 were not used in the analysis because they had a PIC value less than 0.5.

#### 2.5.1. Microsatellite Unweighted Pair-Group Method of Arithmetic Average (UPGMA)

Microsatellites data (bands) for each primer pair from the gel were manually scored into a binary matrix for subsequent calculations. Each polymorphic band was regarded as a single allele and was scored for the presence (1) of the character and absence (0) of the character. The degree of genetic diversity was calculated based on the loci that are polymorphic. The binary data was then entered into Primer-E 5 for Windows (Plymouth Routines in Multivariate Ecological Research) software package [11] for similarity calculations and cluster analysis. The Bray-Curtis similarity method calculations were used to obtain similarity coefficients after which a phenogram was generated by Unweighted Pair-Group Method of arithmetic Average (UPGMA) clustering algorithm.

#### 2.5.2. Ordination of SSR Data

Ordination was done using Principal Coordinate Analysis (PCoA). The PCoA was used to analyze microsatellites data using NTSYS-pc (Numerical Taxonomy and Multivariate Analysis System) software package [12]. The ordination plots for SSR were generated from SSRs data and used to identify trends and patterns and assess the robustness and validity of the clusters generated from UPGMA.

#### 2.5.3. Morphological and Molecular Consistency of Clusters

Unprocessed morphological data was obtained from the Gene Bank and processed in order to evaluate the relatedness of the* P. glaucum* accessions based on morphology analysis and to superimpose morphological characters onto the molecular tree. Out of the ninety-six (96)* P. glaucum* accessions used in this study, only twenty-nine (29)* P. glaucum* accessions have been morphologically characterized. Hence, the remaining sixty-six (66) accessions of the present study are among the morphologically uncharacterized accessions. The characters considered for analysis were early vigour, tillering altitude, number of nodal tillers, plant aspects, lodging susceptibility, fodder potential, spike shape, spikelet shattering, bristle length, and spike density. Nevertheless only discrete morphological data was used in this study. A similarity matrix was constructed using Primer-E 5 for Windows (Plymouth Routines in Multivariate Ecological Research) software package described by Clarke and Gorley [11] for similarity calculations based on the ten (10) discrete morphological characters mentioned above. The Bray-Curtis similarity method was used and a phenogram was generated based on UPGMA. SSRs phenograms were then constructed based on the twenty-nine (29) accessions that had corresponding morphological characterization data in order to investigate the relationship between the morphological characterization data derived cluster and the SSR data derived clusters of the twenty-nine (29)* P. glaucum* accessions. Ordination was then done using Principal Coordinate Analysis (PCoA) on the discrete morphological data and SSRs data in order to compare the number of respective groupings formed by the two datasets and to confirm groupings with the clusters generated by UPGMA.

## 3. Results

### 3.1. SSR Genetic Diversity Calculations

The genetic diversity of the ninety-six (96)* P. glaucum* accessions was calculated using the Shannon diversity index based on SSR primers: 3005, 3035, and 3039 (Figure 3). These primers detected a total of 6 alleles designated a1, a2, a3, a4, a5, and a6. The calculated value of the Shannon diversity index was found to be 0.45 (Table 4) indicating a low level of genetic diversity among the accessions. The Shannon diversity index values are usually between 1.5 and 3.5 in most ecological studies, and the index is rarely greater than 4 [13].

### 3.2. SSR Clusters

**Figure 1:**
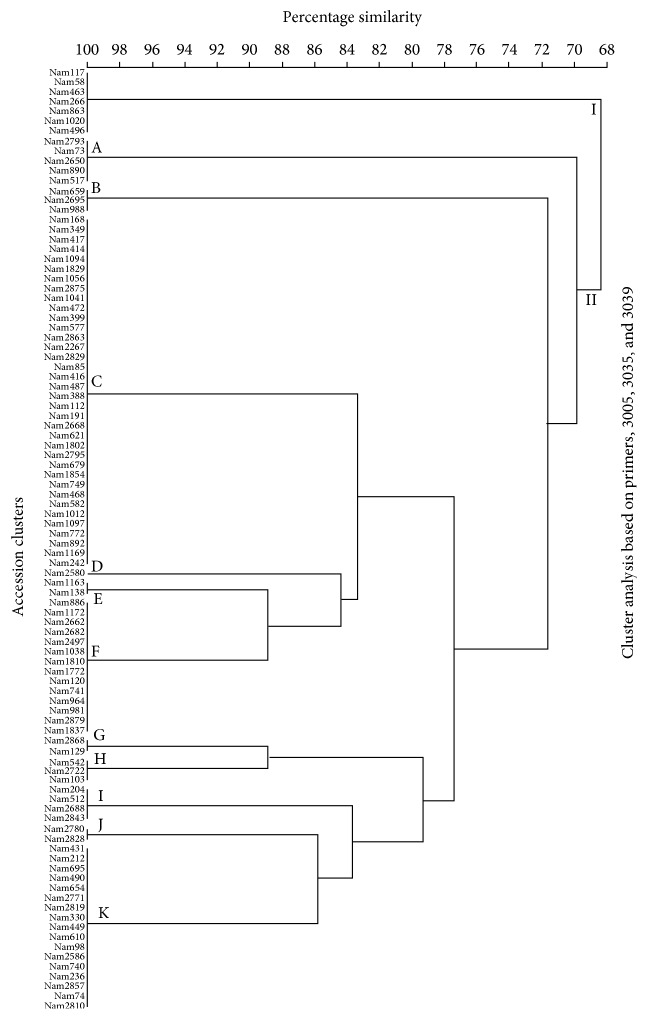
UPGMA phenogram generated from 3005, 3035, and 3039 SSR profiles of 96* P. glaucum* individuals. The scale represents Bray-Curtis similarity coefficients.

UPGMA-generated phenogram (Figure 1) computed using the Bray-Curtis similarity revealed a similarity level of 68%; the 96* P. glaucum* genotypes were divided into two main clusters labeled I and II. The first main cluster was composed of only one (1) cluster while the second main cluster was divided into eleven (11) subclusters designated A-K, respectively.

### 3.3. SSR Data Ordination

The results from PCoA based on SSR markers revealed diversity of the* P. glaucum* by assessing six alleles that were detected. The ordination plot revealed twelve (12) clearly distinct groupings as also indicated in the cluster analysis.

### 3.4. Morphology and Molecular Clusters of 29 Accessions

### 3.5. Cluster Analysis: Morphological Data

*(a) Morphology UPGMA Clustering of Selected 29 Accessions*. The morphological data generated phenogram of the twenty-nine (29)* P. glaucum* landraces displayed the occurrence of two (2) main clusters at 78% similarity level. These clusters were designated (I) for the first main cluster and (II) for the second main cluster. Accessions in subclusters 5 and 6 possess an intermediate fodder potential while accessions in subclusters 7, 8, 9, 10, 11, and 12 possess a good fodder potential. Subclusters 13 and 14 had accessions with a low lodging susceptibility. Accessions in subclusters 15 and 16 possess a conical spike shape whereas accessions in subclusters 17, 18, 20, and 21 possess a candle spike shape. Subclusters 22 and 23 had accessions possessing a compact spike density while subclusters 25, 26, and 27 had accessions with high early vigour.

*(b) Morphology Data Principal Coordinate Analysis of Selected 29 Accessions*. The results from PCoA based on morphological data of 29* P. glaucum* accessions revealed diversity (Figure 4). The ordination plot revealed twenty-eight (28) clearly distinct groupings and confirmed consistency with those obtained from UPGMA cluster analysis.

#### 3.5.1. Cluster Analysis: SSR Data

*(a) SSR UPGMA Clustering of 29 Accessions*. An SSR phenogram (Figure 5) was drawn based on the same twenty-nine (29)* P. glaucum* landraces used for morphology cluster construction. The results showed that the 29 accessions could be grouped into two main groups using SSRs. The two (2) main clusters formed at a similarity level of 69% and were designated (I) for the first main cluster and (II) for the second main cluster. The first main cluster (I) had accessions with an intermediate spike density while subcluster 1 had accessions possessing a candle spike shape. Accessions in subcluster 2 possess a compact spike density whereas subcluster 3 had accessions with an intermediate tillering attitude. Subcluster 4 had accessions with an erect tillering attitude while subcluster 7 had accessions with low lodging susceptibility.

*(b) SSR Data Principal Coordinate Analysis of 29 P. glaucum Accessions*. The results from PCoA of 29* P. glaucum* accessions based on SSR markers disclosed diversity of the* P. glaucum* by assessing six alleles that were detected. The ordination plot revealed eight (8) clearly distinct groupings. The groupings of PCoA confirmed the clusters generated by UPGMA analysis.

## 4. Discussion

### 4.1. SSR Analysis

Only reproducible and scorable bands were used for analysis and interpretation of data in the present study. To ensure reproducibility of the bands, the same template DNA was amplified in 3 different amplification reactions using the same primers. Only strong and reproducible bands were considered for further analyses. However, the unpredictable occurrence of null alleles may have possibly altered the observations on the gels; this could have had an implication on the low level of genetic diversity observed. Null alleles have been found to present problems in the interpretation of results in various studies [14]. Null alleles are alleles that do not give a polymerase chain reaction product. According to Varshney et al. [14], null alleles were revealed by EST-SSR markers in studies on kiwifruit [14], rice [15], spruce [16], and wheat [17, 18]. Furthermore, Varshney et al. [14] describe the incidence of null alleles pertaining to microsatellites as a consequence of the deletion of microsatellites at specified locus and mutations (indels or substitutions) in the primer binding sites. This may result in the wrong interpretation of information because heterozygotes cannot be identified and reaction failures cannot be detected. Even though SSR analysis on the 1.5% agarose gels was good enough to determine genetic diversity, the use of polyacrylamide would still be good.

### 4.2. SSR Genetic Diversity Calculations

The results from the PIC of the primers used for analysis in present study indicated that the primers provided a significant amount of information regarding polymorphism. On this basis, they were used for genetic diversity calculations using the Shannon diversity index. The Shannon diversity index revealed a low level of diversity. It can also be argued that the low level of polymorphism achieved would be somewhat expected because the primers used in the present study are EST derived. Literature [16–23] has revealed that EST-SSR primers are less polymorphic in crop plants compared with genomic derived SSRs due to better DNA sequence conservation in transcribed regions. The Shannon diversity index value (0.45) obtained is regarded as low in comparison to those obtained in literature [13, 24] arguing that the Shannon diversity index values usually range between 1.5 and 3.5 of which the values obtained in the present study were lower than 1.5. The results of the present study therefore suggest that the selected* P. glaucum* genotypes have narrow genetic diversity, despite the high PIC value of primers used.

### 4.3. SSR UPGMA Clusters

The results from the SSR cluster analysis showed that the genotypes could only be grouped into two main clusters which were formed at a similarity level of 68%. This level of similarity was relatively high indicating a low level of genetic diversity among the Namibian* P. glaucum* germplasm. However, it can be speculated that assessing genetic diversity of the current* P. glaucum* genotypes sampled directly from the fields of different regions would display a higher level of genetic diversity accountable to possible mutation events that would have taken place from the time the accessions were collected (most accessions were collected in 1991). This is because the implications of mutation and migration play a huge role in the restoration of the genetic diversity and the influences of these two factors seem to be highly reduced in seed multiplication experiments that are routinely carried out at the NPGRC (National Gene Bank of Namibia).

Other studies [4, 25] also argued that mutation and migration and their interaction with selection have six important implications in conservation genetics: (1) the regeneration of genetic diversity due to mutation, (2) recovery of genetic diversity by migration, (3) migration often reversing inbreeding depression, (4) the impact of gene flow (migration) from related species (introgression), (5) maintenance of genetic diversity through mutation and migration, and (6) the load of deleterious alleles in population; this is caused by the balance between deleterious mutation and selection results in an ever present but changing pool of rare deleterious mutations (mutation load) in populations. In addition, the SSRs clusters did not form on the basis of the region of origin of the accessions but were mixed within the groupings. This could possibly be attributed to the wide germplasm exchange that occurs between villages and could even extend among regions because some regions are the main producers of the crop compared to others. Hence these regions could also have been the source of germplasm for other regions. This suggestion is supported by both studies [5, 26] that observed that famers acquired 80% of their seeds from their neighbors and approximately 60% of the farmers travel long distances in order to exchange or buy seeds in quest of maintaining the vigour of the crop. However, the study [5] argued that a high genetic diversity would be expected in landraces and open pollinated varieties of* P. glaucum* because pearl millet is naturally outcrossing. In addition, it was further suggested [5] that genetic diversity is certainly lost in inbreeding depressions particularly through seed multiplication experiments that occur in a closed system. Comparable results to the present study were also obtained [27] by comparing the amount of alleles between wild* P. glaucum* accessions and cultivated* P. glaucum* accessions. Their findings indicated lower allelic richness in the cultivated* P. glaucum* compared to the wild type. Their study also revealed that the cultivated accessions displayed the presence of less alleles (23%) in relation to the wild type. In addition, they found an average gene diversity of 0.49 in the cultivated compared with 0.67 in the wild type. This gave an overall gene diversity of cultivated* P. glaucum* that is 28% lower than in the wild* P. glaucum*.

### 4.4. SSR Ordination

**Figure 2:**
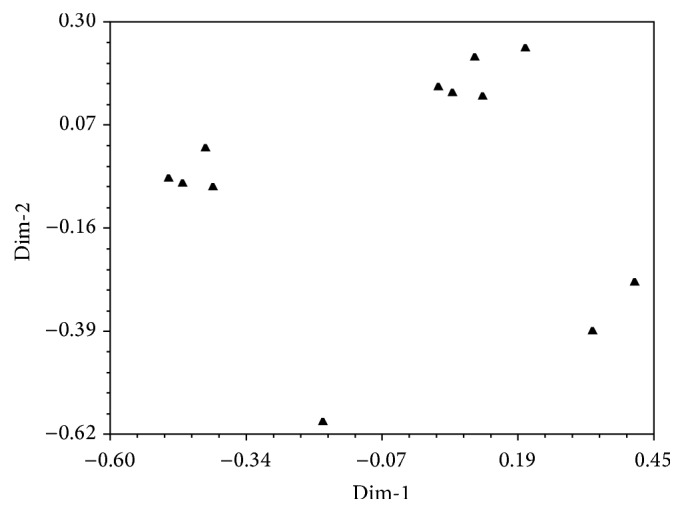
Principal Coordinate Analysis of the 96* P. glaucum* genotypes based on SSRs marker data.

**Figure 3:**
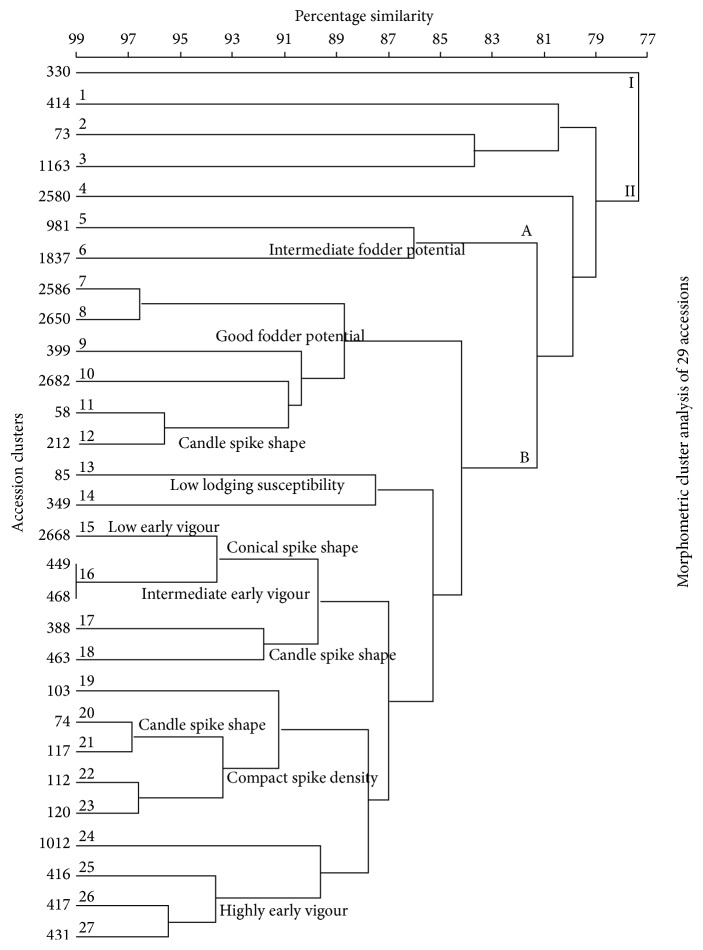
UPGMA phenogram constructed from morphometric data showing some phenotypic traits of selected 29* P. glaucum* individuals. The scale represents Bray-Curtis similarity coefficients.

**Figure 4:**
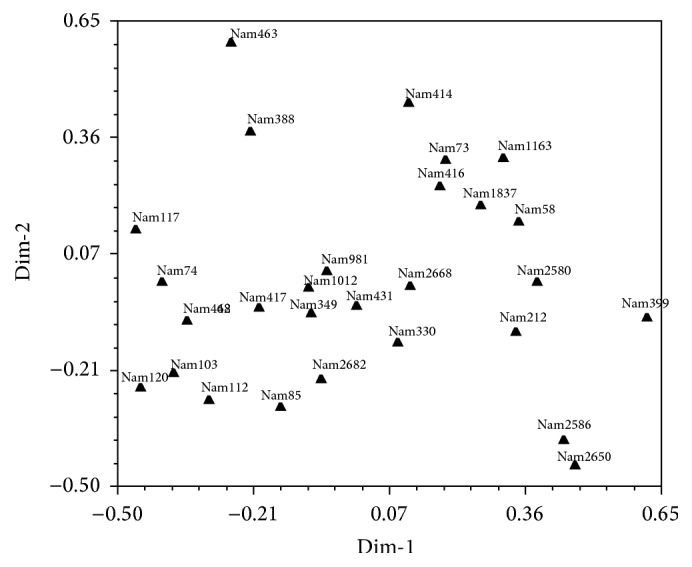
Principal Coordinate Analysis of the 29* P. glaucum* genotypes based on morphological data.

**Figure 5:**
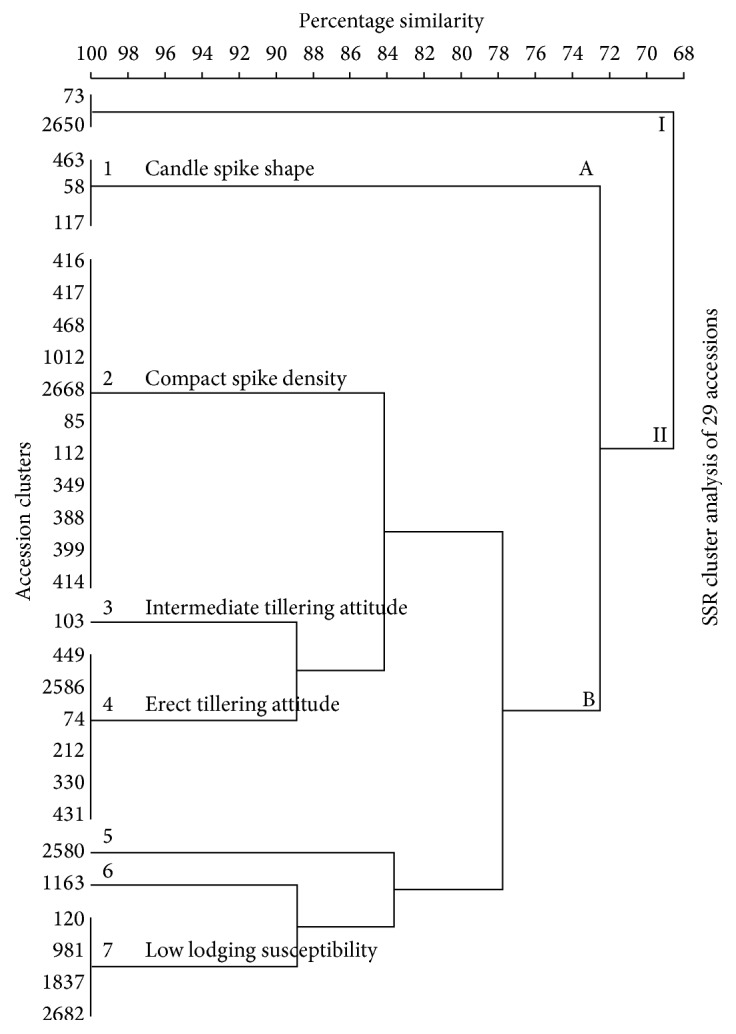
UPGMA phenogram constructed from 3005, 3035, and 3039 SSR profiles showing some phenotypic traits of 29* P. glaucum* individuals. The scale represents Bray-Curtis similarity coefficients.

PCoA was employed on the six alleles (see Figure 2) that were identified by SSRs from the 96* P. glaucum* accessions. In comparison to SSRs results that produced 12 clusters from the 96 accessions analyzed based on UPGMA, 12 groupings were produced when the same 96 accessions were analyzed using PCoA. This suggests that the clusters initially generated using UPGMA were reliable and furthermore supports the argument that the use of the SSRs technique in such a study is reliable. However, previous studies have used Principal Component Analysis (PCA) to assess genetic diversity mostly between wild and cultivated accessions [27, 28]. It was revealed [28] that wild and cultivated accessions demonstrated a comparable distribution in the PCA while another study [27] found a noticeable variation between accessions of wild* P. glaucum* which displayed unique allele combinations and cultivated* P. glaucum* which revealed homogeneity. Similarly, a clear variation between accession groupings was achieved by PCoA in the present study. However the study revealed a low level of genetic diversity based on SSRs compared to that obtained previously using isozymes [29, 30]. This discrepancy could be due to the different evolutionary properties of SSRs and isozyme loci.

### 4.5. Morphology and Molecular (SSR) Clusters of Selected 29 Accessions

**Figure 6:**
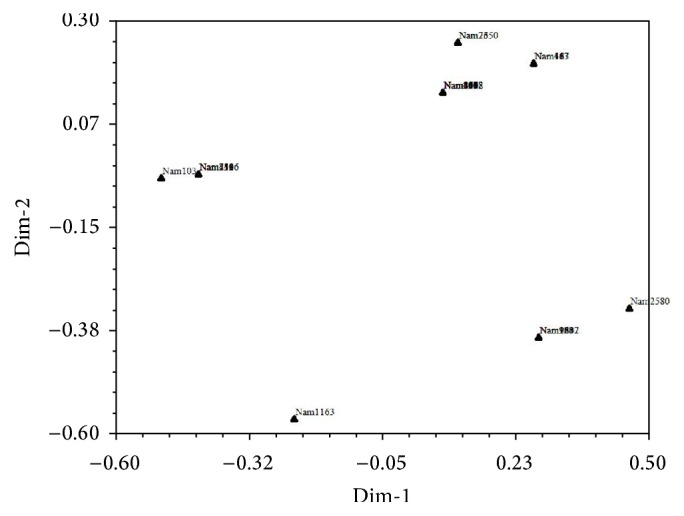
Principal Coordinate Analysis of the selected 29* P. glaucum* genotypes based on SSR data.

In order to link the phenotypic variation to the genotypic variation, 29* P. glaucum* genotypes were clustered based on UPGMA using SSRs and morphological data (Figure 6). SSRs markers have been employed in the integration of genetic, physical, and sequence-based physical maps in plant species. This has enabled breeders and geneticists to link phenotypic and genotypic variation [31].

Morphological data UPGMA clustering of 29 accessions revealed the formation of two main clusters at 78% similarity level while the UPGMA clustering of SSR data displayed two main clusters at a similarity level of 69%. In addition, the similarity level (69%) and cluster B of the SSR data phenogram were closely related to those of morphological data with a 78% resemblance. The minor variations in the particular accessions found in clusters of the SSR phenogram in comparison to the observed morphology clustering based on the ten selected discrete traits are possibly a consequence of few number of selected traits in the present study and environmental factors such as soil composition, temperature, and rainfall that influence the resulting morphology (appearance) of plants [32–43].

The morphology data phenogram disclosed that clustering of the respective accessions was as a result of possessing similar traits of fodder potential, lodging susceptibility, early vigour, spike density, and spike shape. There were some overlaps in the observed traits in the tree which is probably because some genetic characteristics occur along a continuum. That is, a trait can strongly, moderately, or weakly be expressed in the same genotypes. It has been argued [44] that variable expressivity occurs when variability in the level of phenotypic expression is observed from the same genotype. From the SSR phenogram of the 29 morphologically characterized genotypes, four traits were clearly identified upon which some clusters were formed. This suggests that these traits are strongly expressed in the genotypes under these clusters. Lodging susceptibility, tillering attitude, spike density, and spike shape were identified as the strongly expressed characters. Lodging is the permanent dislocation of stems from the erect position. Lodging is mostly problematic in times of heavy rain and strong winds. Lodging has been a problem in cereals for a long time especially after storms that leave the whole fields of cereals flattened [45]. However, it has been argued [46] that lodging resistance can be improved by selecting amongst the progeny plants for shorter and solid stems.

## 5. Conclusions and Recommendations

The genetic diversity of Namibian* P. glaucum* germplasm was detected by analyzing the SSRs molecular markers. It was revealed that the Namibian* P. glaucum* germplasm has a low genetic diversity. Furthermore it was disclosed that the microsatellites technique was informative on the basis of clusters corresponding to morphological data clustering. The SSR phenogram showed a consistency above 70% with the morphology data phenogram. This suggests that SSR clusters may well be used to identify* P. glaucum* genotypes and predict the phenotype of uncharacterized genotypes which is useful in resource management for germplasm conservation and breeding programs. The NPGRC will only store the accessions that are diverse; this will reduce the cost of storage incurred because duplicated accessions will be discarded and only genetically diverse accessions will be stored. Furthermore, there was no noticeable diversity pertaining to the regions of origin of the accessions; this indicated a low level of genetic diversity among the Namibian germplasm.

The findings of this study support the recommendation that efforts must be made to widen the available gene pool and germplasm in order to increase the genetic diversity of* P. glaucum.* In addition, the germplasm must be evaluated from time to time to investigate the level of genetic diversity using additional techniques such as RAPDs and ITS. It is further recommended that a similar study should be carried out using recent collected accessions and the number of accessions in the analysis must be increased due to some inherent limitations that occur in the sampling methods. Moreover, there is a need for molecular characterization of all the accessions in the Namibian germplasm collection in order to select the most divergent genotypes which can be used in hybridization programs with the intention of improving the crop genetically. It is also imperative to incorporate farmers in an attempt to conserve and improve the crop (*P. glaucum*) because they also play a role in the maintenance of the gene pool in the country. It appears that offering agricultural training or workshops to farmers on the conservation of genetic resources would help solidify the advancement in the crop. This training will allow participatory plant breeding in which farmers and plant breeders work together to develop plant varieties.

## Figures and Tables

**Table 1:** Details of SSR primers developed from pearl millet EST sequences that were used in present study.

Name	Forward primer sequence (5′-3′)	Reverse primer sequence (5′-3′)	Repeat
3002	AAAGTTACCGGGAGGGTAAAAA	TCGCCTAAAAACTGGAGGAA	AAAC(3)
3005	CGCGGTGTTCTCACACAC	TGTGAATTCCGCGGGTATAG	AC(14)
3006	AAATCGGTCGTGGTGAAGTT	GAGAATGTGGGAGACACACG	AC(16)
3009	CTGTACCATGTGCGCTGATT	GCGCATATATGTGGGTGTGT	AC(16)
3011	CACGCCCTTTTTACCTTGAC	CGCGACACGTCCTACACTAA	AC(21)
3013	TGTGGGAGAGAGGAGAGTCC	CGCGAGATGATGTGTGGT	AC(33)
3014	TGCTTCACAGCCTCTCCATA	CCACCATGCAACAGCAATAA	ACC(8)
3016	TTGTGGCTGAAGAAGAGATCC	AATGTGGGGAGAGACACACG	CA(17)
3017	CACCAAACAGCATCAAGCAG	AGGTAGCCGAGGAAGGTGAG	CAG(7)
3018	CGATGACACCTGTGCGTATT	ATCGAACTGCACGTTAGCAA	CATG(4)
3019	GCGCACCACCTGTGTCTAT	CATGCAGAGAAAAATCAAGCA	CGTA(4)
3020	GTTCCATGGAGCTGGAAGTC	GCTAGAACAGGGCCGTTACA	CGTG(5)
3021	GCCGACAGGAAGATTACGAT	AGCAAAACGCAGAACAACAG	CGTG(5)
3022	CTGGAAGTCCTTCTCGGTTG	CTGCTCCGCTCTGAATCTG	CGTG(5)
3025	GTTGCAGATGAGCGATCGTA	AGCGCAAAGAGTGTAACTTGG	CTC(6)
3026	GTGAGGCCTCGAACAAACAC	GCCGACCAAGAACTTCATACA	CTC(6)
3027	ACACCATCACCGACAACAAA	AGTGACCTGGGGTACAGACG	GAT(6)
3028	ACGATTCTTCGTCGTTCCAG	ATACGATACGCGCGAGCTAC	GATC(4)
3029	ACCAGCAACAGCAGCAGAG	ACACACTGCGACAAGTGGAG	GCA(6)
3032	AGGTAGCCGAGGAAGGTGAG	CAACAGCATCAAGCAGGAGA	GCT(8)
3033	GAGGGCCAGCTCTCCTAGAT	CCCTAACCACAGAGGGACAC	TGCC(4)
3035	GCCAAGGAGGTCAAGATCG	ACACGACTCGACTCAGACCA	TGCC(4)
3037	CGTCGCTGCTCTTTCTTCTT	ATTTCAGAAACGGCAACCAA	TGGA(4)
3038	CTCTCGGTTTGACGGTTTGT	GGGGAAAACAAAGTTGCTCA	TGT(6)
3039	GGCACGAGGGGCTAAGTAA	GGAACGCCGAGTACACAGAT	TGT(6)

**Table 2:** The calculated sample size from the total accessions collected from each region.

Region in Namibia	Total accessions per region	Regional sample size
Karas	15	1
Zambezi	292	19
Okavango (East + West)	716	48
Otjozondjupa	20	1
Oshikoto	28	2
Kunene	29	2
Omusati	144	10
Ohangwena	175	12
Oshana	22	1

*Total values*	*1441*	*96*

**Table 3:** PIC values of the SSR primers: 3005, 3020, 3039, 3016, and 3035.

Primer	PIC value
3005	0.64
3016	0.48
3020	0.48
3035	0.76
3039	0.69

**Table 4:** SSR Shannon diversity index calculated from primers 3005, 3035, and 3039.

Allele	Frequency (*P*_*i*_)	*P*_*i*_log⁡*P*_*i*_	*H* = − ∑_*i*=1_^*K*^ *p*_*i*_log⁡*p*_*i*_
a1	0.29	−0.16	0.45
a2	0.92	−0.03	
a3	0.93	−0.03	
a4	0.05	−0.07	
a5	0.93	−0.03	
a6	0.18	−0.13	
